# Fit 4 surgery, a bespoke app with biofeedback delivers rehabilitation at home before and after elective lung resection

**DOI:** 10.1186/s13019-019-0951-6

**Published:** 2019-07-05

**Authors:** Salma Bibi Kadiri, Amy Pamela Kerr, Nicola Katy Oswald, Alina-Maria Budacan, Sarah Flanagan, Christopher Golby, Stuart Lightfoot, Babu Naidu

**Affiliations:** 1 Department of Thoracic Surgery Research, Heartlands Hospital, Bordesley Green East, Birmingham, B9 5SS UK; 2 Institute of Inflammation and Ageing, College of Medical and Dental Sciences, Centre for Translational Inflammation Research, University of Birmingham Laboratories, Queen Elizabeth Hospital Birmingham, Edgbaston, Birmingham, B15 2TT UK; 3 Institute of Applied Health Research, University of Birmingham, Edgbaston, B15 2TT UK; 4 Evolyst- The Innovation Centre, Warwick Technology Park, Gallows Hill, Warwick, CV34 6UW UK

**Keywords:** Pulmonary rehabilitation, Lung Cancer, Exercise, Thoracic surgery, Quality of life, Intervention, Technology

## Abstract

**Background:**

Pulmonary rehabilitation programme for lung surgery patients can reduce the risk of post-operative complications but compliance to programmes can be limited by access to health care. We developed a home-based rehabilitation app and tested its feasibility in patients undergoing lung resection surgery.

**Methods:**

A cohort study was conducted over 18 months at a regional thoracic unit. The Fit 4 Surgery app included ten exercises. Patients were instructed to exercise for at least three minutes for each exercise. Data was transmitted back to the researchers remotely. Data was also collected from a contemporaneous group of surgery patients who attended local outpatient-based Chronic Obstructive Pulmonary Disease rehabilitation classes. Quality of Life and outcomes data in the app group were collected. Patients were also interviewed about their experience of the app.

**Results:**

App patients had a shorter wait before surgery compared to patients attending rehabilitation classes (24 vs 45 days) but managed four times as many sessions (2 vs 9), improving incremental shuttle walk test distance by 99 ± 83 (*p* < 0.05) metres before surgery. Five themes were gathered from the interviews.

**Conclusion:**

An app based programme of rehabilitation can be delivered in a timely fashion to lung surgery patients with demonstrable physiological benefits; this will need to be confirmed in further clinical trials.

**Clinical trial registration number:**

ISRCTN00061628. Registered 27 May 2011.

**Electronic supplementary material:**

The online version of this article (10.1186/s13019-019-0951-6) contains supplementary material, which is available to authorized users.

## Background

Up to 15% of patients develop post- operative complications after lung surgery which can result in death, admission into an intensive care unit, prolonged hospital stay and readmission to hospital after discharge [1, 2]. The evidence on pre- and post-operative rehabilitation in lung resection surgery is poor, mainly due to the heterogeneity of patient population, interventions and outcomes [3]. Recently published Enhanced Recovery After Surgery guidelines recommend the use of prehabilitation for patients with borderline lung function or exercise capacity [4]. Meta-analysis of studies of pulmonary rehabilitation and/or exercise classes for lung surgery patients demonstrate improvement in exercise capacity, quality of life and a reduction in post-operative complications [5, 6]. However, access to rehabilitation services can be hampered because of limitations in local health care resources and patient reluctance to attend multiple classes. Rehabilitation services can find it difficult to deal with the fluctuation in demand from flow of lung cancer surgery patients so can struggle to provide a consistent service [7].

Therefore, there is a need for a service that can be delivered immediately at the convenience and in the control of the patient. Mobile device use in the healthcare sector to access systems has rapidly evolved with an explosion in software applications (apps). Apps have been shown to support education for self-management and provide extensive feedback to users to facilitate behaviour change [8, 9]. Physical activity interventions incorporating technology have been used with varying degrees of success in the medical disease setting but not in the surgical arena [5]. Knowing that pulmonary rehabilitation consisting of exercise classes is beneficial to lung surgery patients but is resource limited and that apps can promote physical exercise in other disease settings, we sought to develop a bespoke pulmonary rehabilitation app and test its feasibility and acceptability to patients undergoing lung resection surgery.

## Methods

An ethically approved cohort study (Research Ethics Committee reference 10/H1208/41) was conducted over 18 months at a regional thoracic unit. Inclusion criteria were broad; any patient deemed eligible for curative lung cancer surgery based on British Thoracic Society guidelines by the multidisciplinary teams referring to a regional thoracic surgery unit.

Surgery was never delayed for the purposes of the study, thus time spent using the app varied. Patients recommenced the programme 2 weeks after discharge. Data on adherence to local COPD pulmonary rehabilitation classes was also collected from a contemporaneous group of patients, who had agreed to attend these sessions twice a week up to the day of their surgery and for 6 weeks following surgery as described previously [7]. The only comparison made between the two groups pertained to the process measures (ie: number of sessions pre and post surgery).

### Intervention

The ‘Fit 4 Surgery’ app consisted of ten exercises, both upper and lower limb, aerobic and strength, based on the lung cancer ‘Rehabilitation for Operated lung Cancer’ surgery (ROC) programme and available on the patient website (http://www.thoracicsurgery.co.uk) [5].

The app was developed for an Ipad mini 2 cellular (Apple Inc. California, USA), configured with a blue tooth enabled pulse oximeter (Creative PC-68B, Shenzhen Creative Industry Co. Ltd., China) and a Subscriber Identity Module (SIM) card to enable wireless feedback of data to the researchers. The app collected baseline measurements of oxygen saturation and heart rate for safety. It provided the patient with a target heart rate (> 60% of maximum heart rate) based on their age to be achieved during each exercise and patients were given all equipment.

**Fig. 1:**
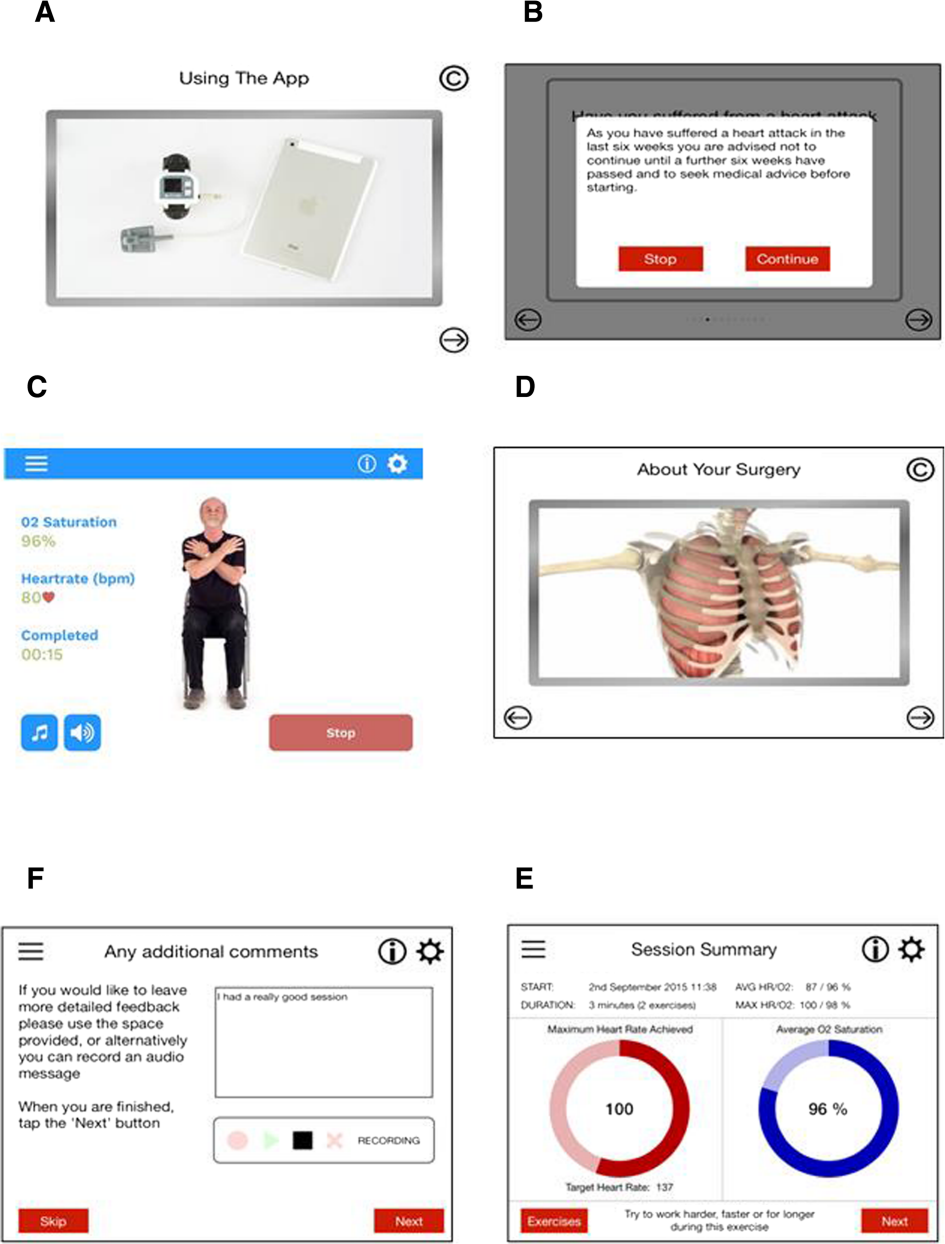
Screenshots from Fit 4 Surgery App. **a** training video for patients on how to use the app. **b** safety notification screen (c) one of the exercise videos which shows feedback e.g. duration of exercise, heart rate and O2 saturation. **d** one of the education videos that patients can view using the app. **e** a summary screen post exercise which shows if target heartrate was achieved, average O2 saturation and motivation feedback. **f** Additional comments box screenshot

Each exercise was demonstrated to the patient through a series of video clips on the app which they could follow whilst the pulse oximeter collected heart rate and oxygen saturation levels continuously (Fig. 1).

The patients were instructed to exercise for at least three minutes per exercise. After each exercise, a summary screen provided feedback to patients on how effective they were at completing the exercise. This included: duration of exercise, average oxygen saturation and whether they reached their target heart rate. At the end of each exercise patients could rate their effort by completing a Borg scale breathlessness questionnaire on the app [10].

At the end of each session, a summary of all exercises completed in that session was displayed. Patients completed a series of questions rating their overall exercise session and could leave any further comments through an audio recording captured through the ipad. Anonymous data from the app was transferred wirelessly on to a cloud based server compliant with National Health Service (NHS) information governance guidance. Data from this server could be accessed by the research team. Videos from the ROC education programme were incorporated into the app, to inform patients about the surgery, importance of exercise; patient pathway [7]. Patients in the contemporaneous rehabilitation class group also received this information by a written leaflet and/or reference to the website, and/or provision of a DVD.

### Development

The exercise programme was refined in an iterative process by a stakeholder group consisting of thoracic surgeons, physiotherapists, patient representatives, thoracic surgery nurses, and app developers. This group met at regular time intervals during the project to develop and refine the app and its operations. The app was tested in a hospital pulmonary rehabilitation class with five lung surgery patients and feedback gathered on ease of use and ‘bugs’ in the system. Thus an iterative process of building functionality was followed and feedback incorporated into the second stage of development to refine the performance of the app. The app was created using the Swift 3 coding language and was compatible with the iOS 11 operating. App data was transferred to a RESTful (Representational State Transfer) web service, which allows data to be stored in the cloud, and then accessed through a standard web browser.

### App training

Patients were registered on the app and their baseline heart rate was measured. Registration, in addition to baseline demographics, included a series of questions regarding co-morbidities with generated appropriate pop up messages with contraindications and advice relevant to specific conditions (Fig. 1). Patients were taught how to use the app by the researcher. This was delivered from a scripted training document to ensure consistency. In addition to the one-to-one consultation, patients were given an instruction booklet with details on how to use the app and troubleshooting issues (Additional file 1). Patients were called once a week whilst using the app by the research team to assess if they had any technical problems.

### Contemporaneous ‘rehabilitation class’ group

Patients were enrolled into local COPD rehabilitation classes in a pragmatic fashion; these could take place in the hospital or community based setting and in a group or individual class depending on the services offered locally [5]. All classes followed the national guidance on COPD rehabilitation; they were scheduled twice a week, lasted 90 min and included both strength and aerobic exercises for both the upper and lower body. These exercises were similar to those used in the app. Data on process measures from these classes were retrospectively collated.

### Assessments

Demographic data was collected for the app group as follows: age, COPD, ischaemic heart disease, body mass index (BMI), smoking status, surgery and analgesia type, modified Medical Research Council (mMRC) dyspnoea scale, Eastern Cooperative Oncology Group (ECOG) performance status, spirometry and pathology.

Delivery of the exercise programme, testing usability and evaluation of the improvement was established in a mixed-method approach and performed along the following lines:

#### Process measures

Measures including time taken from identification of patient to first rehabilitation day and number of days of rehabilitation before and after surgery were noted.

#### Physiological parameters

App patients completed an incremental shuttle walk test (ISWT) and spirometry before surgery, pre-and post-rehabilitation. Frequency, type and duration (minutes) of exercises completed using the app in each session were recorded.

#### Patient experience of app and effect on QOL effects / usability

Semi-structured telephone interviews were undertaken with 13 patients by an independent researcher to avoid bias and increase validity of the information collected. Interviews were undertaken 2 weeks after patients finished using the app. All interviews were digitally recorded and transcribed. The transcripts were analysed using content analysis. The following areas of interest were explored with participants: motivation for using the app: effectiveness of staff communication and written communication about how to use the app, usability of the app, impact of the app upon perceived (and actual) levels of fitness, any specific problems encountered using the app and any factors that influenced their use of the app, what aspects of the app were particularly useful and recommendations for changes to improve the app.

Patient quality of life was measured using European Organisation for Research and Treatment of Cancer Quality of Life Questionnaire 30 (EORTC-QLQ30) pre and post rehabilitation pre-surgery and, 6 weeks’ and 5 months’ post-surgery.

#### Outcome measures

Inpatient length of stay, rate of postoperative pulmonary complication (PPC), ITU admissions, hospital length of stay and readmission to hospital within 30 days of surgery were recorded in the app group.

### Statistical analysis

Data presented is summarised using appropriate parametric and non-parametric methods. The only intergroup comparison was made in relation to process measures.

## Results

Characteristics of the app group are shown in Table 1.

**Table 1:** Demographics

	App group (*n* = 31)
Age, mean (SD)	64 (12)
BMI, mean (SD)	25.7 (9.6)
% predicted FEV1, mean (SD)	74.2 (34)
Measured FVC, mean (SD)	3.40 (1.14)
% predicted DLCO,	68.2%
Pathology	
NSCLC	54.8% (17)
Other lung Ca	22.6% (7)
Metastatic	3.2% (1)
Benign	19.4% (6)
Self-reported pre- op activity level % (N)	
Able to walk < 400 m	26% (8)
Able to walk at least 400 m	19% (6)
Able to walk at least 2 km	6% (2)
Able to walk > 2 Kilometres	48% (15)
Smoking % (N)	
Never	19.4% (6)
Ex > 6 weeks	41.9% (13)
Ex< 6 weeks	12.9% (4)
Current	25.8% (8)
Ischaemic heart disease % (N)	6.5% (2)
COPD % (N)	27.6% (9)
mMRC score, median (IQR)	1 (0–1)
ECOG performance status, median (IQR)	0 (0–1)

*BMI* Body Mass Index, *FEV1* Forced expiratory volume, *FVC* Forced lung capacity, *NSCLC* Non-small cell lung cancer, *DLCO* Diffusing capacity of the lung for carbon monoxide, *COPD* Chronic Obstructive Pulmonary disease, *ECOG* the Eastern cooperative oncology group

### Process measures (Tables 2 and 3)

Patients in the app group waited a median of 6 (range 13–33) days to receive the app and completed a median of 9 (range 1–37) sessions of exercises on the app before surgery and a median of 4 (range 1–7) sessions per week. Patients in the class group waited a median of 5 (range 0–23) days to be seen in a rehabilitation class and attended 4 classes (range 1–15). However, 32% [11] of patients did not use the app post-surgery. For the patients who had attended rehabilitation classes’ pre- surgery, 79% did not attend rehabilitation classes post-surgery. Exercise measures are displayed in Table 2. In summary, app patients had a shorter time before surgery (24 vs 45 days) but managed to complete more than 4 times as many exercise sessions (2 vs 9) and completed more sessions after surgery (2 vs 0) than those in the class group.

**Table 2:** Process measures

	App (*N* = 31)	Classes (*N* = 34)
Days from screened to 1st rehab, median (IQR)	6 (2–13)	5 (0–23)
Days from rehab to surgery date, median (IQR)	24 (13–33)	45 (27–71)
Rehab sessions before surgery, median (IQR)	9 (6–13)	2 (1–8)
Days of rehab post-surgery, median (IQR)	2 (0–7)	0 (0–0)

**Table 3:** Exercise parameters in app group

App- exercise parameters	Median (%)	Range
Number of sessions pre-surgery	9	1–37
Number of sessions per week pre-surgery	4	1–7
Total exercise time (mins) pre-surgery	158	3–1226
Time spent in target heart rate of ≥60% (%)	32	3–93
Number of exercises pre -surgery		
Upper body	14.5 (46.3)	1–73
Lower body	21.5 (53.7)	1–71
Median number of sessions post-surgery	2	0–30
Median number of sessions per week post-surgery	< 1	0–4
Total exercise time (mins) post-surgery	22	0–888
Median Number of exercises post-surgery		
Upper body	4 (47)	0–90
Lower body	5 (53)	0–87

### Physiological parameters

**Fig. 2:**
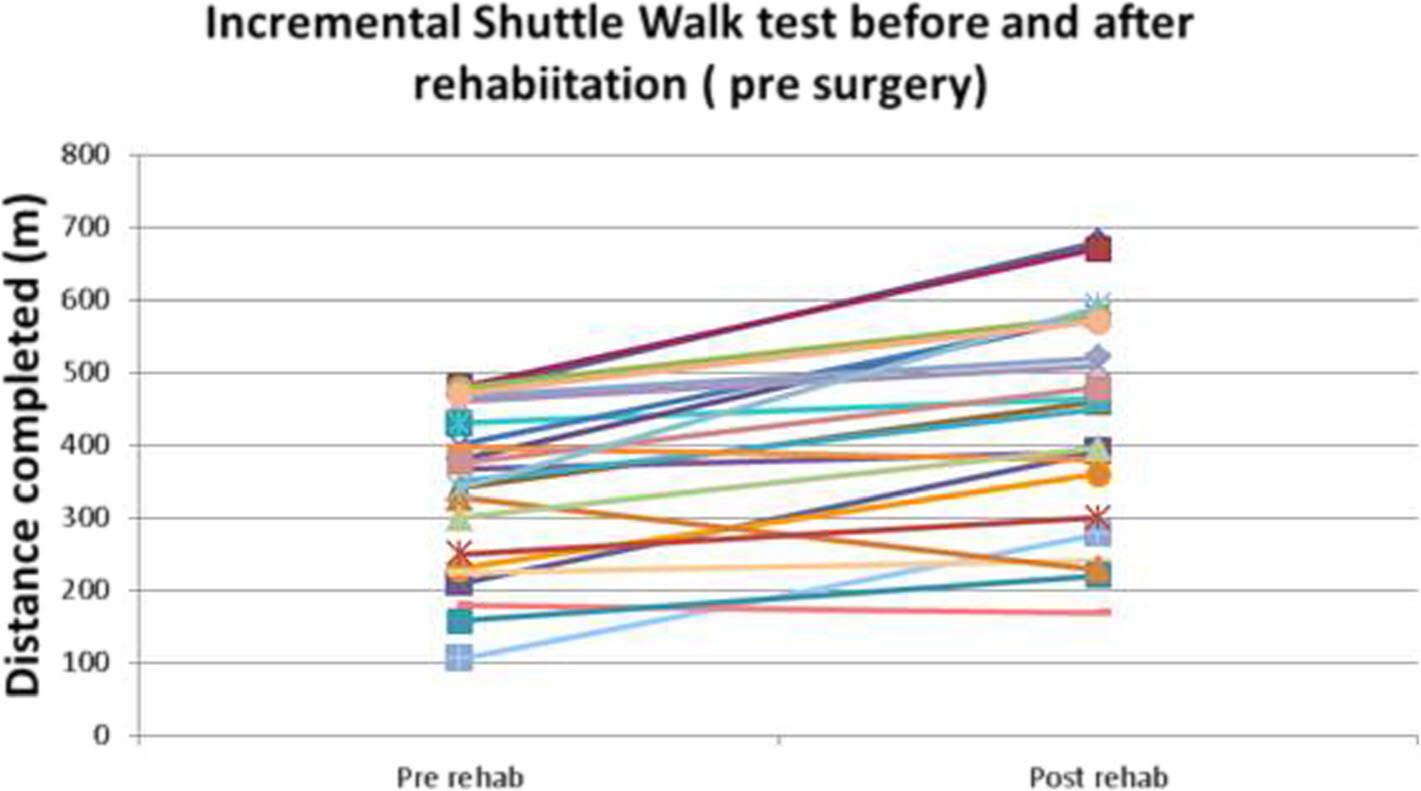
Incremental shuttle walk test before and after rehabilitation in app group. Each patient is represented by one line

Baseline median ISWT in the app group was 367 m (IQR105–480) prior to rehabilitation and improved to 450 m (IQR169–680) following app rehabilitation, prior to surgery (Fig. 2). There was no relationship between magnitude of increase in the ISWT and total exercise time or time spent at target heart rate during exercise or any other exercise parameter.

### Patient experience of app and effect on QOL effects / usability (see Fig. 3)

Interviews were conducted until saturation was achieved (*n* = 13). Seven females and six males were interviewed.

**Fig. 3:**
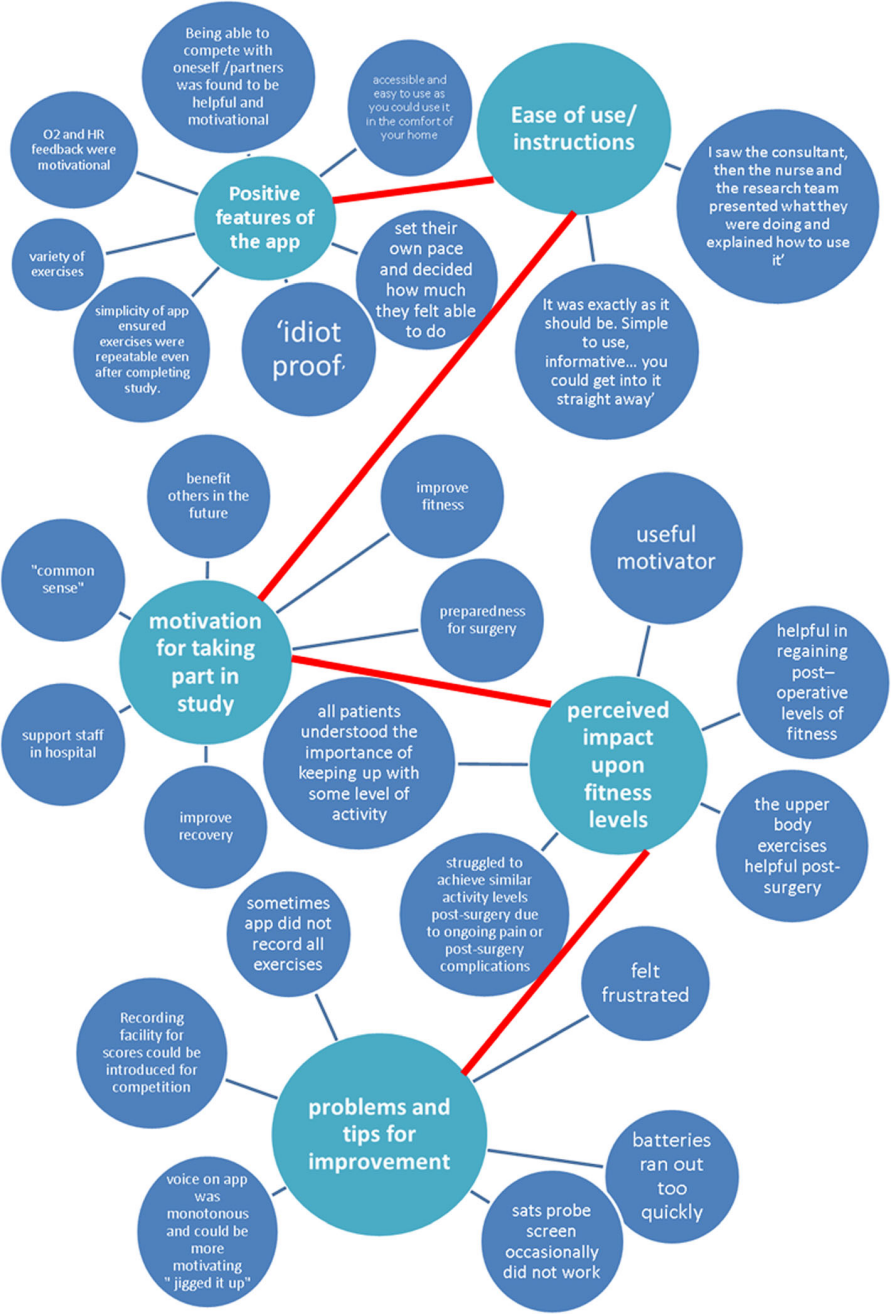
Map to demonstrate the qualitative themes and quotes gathered from patient interviews

#### Motivation for taking part in the study

There were six motivations for patients’ participation in the study.

#### Ease of use/instructions to use the app

All the patients found the verbal and written communication, straightforward. They felt that the research team fully explained how to use the app. All but 3 patients had some experience of using digital technologies to varying degrees (from using a smartphone to using smart devices for texting and using apps). One patient had experience as a web developer and was impressed with the app. Other patients with less experience also found it easy to use.

#### Reported positive aspects and features of the app

The app enabled patients to set their own pace. The simplicity of the app meant that the exercises were repeatable. Many patients found that being able to see their oxygen levels and heart rate was motivational and the variety of exercises was also welcomed. The novelty factor of using the app for exercise was appealing to some patients and even patients who had good levels of fitness prior to using the app found it beneficial.

#### Problems and tips for improvements

Despite the overall ease of use, patients identified some minor areas that may improve patient experience and utility of using the app and these were incorporated into the app on subsequent iterations.

#### Perceived impact upon fitness levels

Patients had varied levels of fitness prior to surgery but overall, almost all reported benefit from using the app. Success seemed to be contingent upon both the ease of use, personal levels of motivation and health status.

#### Quality of life scores (Table 4)

All of the scales and single-item measures ranged in score from 0 to 100. A high scale score represented a higher response level; a high score in the functional scale is classed as a healthy level of functioning, a high score for a symptom item represents a greater level of symptomatology. A change in any scale of at least 10 is considered clinically relevant [10]. The changes in scores pre surgery to 5 weeks post-surgery to 5 months post op in the app group are shown in Table 4. Of note, the Global Health score at 5 months for the app significantly increased and had returned to baseline level.

**Table 4:** EORTC QLQ C30 scores in the app group

Domain	Change in scores pre surgery to 5 weeks post-surgery	Change in scores post op 5 weeks to 5 months
Physical	19.0 (17.8)	−4.73 (27.7)
Role	19.3 (30.2)	−4.98 (41.5)
Fatigue	−23.4 (26.4)	3.88 (35.2)
Pain	−18.4 (33.2)	15.7 (32.6)
Dyspnoea	−26.8 (28.0)	1.7 (42.7)
Global Health status	19.6 (19.1)	−10.0 (29.8)

Mean (standard deviation) change in QOL scores from pre rehabilitation to 5 months post-surgery

### Outcome measures (Table 5)

Inpatient length of stay, rate of postoperative pulmonary complication (PPC), ITU admissions, hospital length of stay and readmission to hospital within 30 days of surgery were recorded in the app group. 48.4% of patients underwent lobectomy and the same percentage benefited from a sublobar resection, either via VATS (54.8%) or thoracotomy (45.2%). The median length of stay was 4 days and the PPC rate was 9.7%.

**Table 5:** Outcome measures

	App (*N* = 31)
Surgery % (N)	
Lobectomy	48.4% (15)
Sublobar	48.4% (15)
Pneumonectomy	3.2% (1)
Analgesia % (N)	
Epidural	41.9% (13)
Morphine infusion	6.4% (2)
Paravertebral	51.6% (16)
Surgical technique % (N)	
Thoracotomy	45.2% (14)
VATS	54.8% (17)
Length of stay, median (IQR)	4 (3–7)
PPC rate % (N)	9.7% (3)
Unplanned ITU rate % (N)	6.4% (2)
Unplanned ITU LOS in days’ median (IQR)	1.5 (1–2)
30 day hospital readmission % (N)	9.7% (3)
30-day mortality % (N)	0% (0)

*PPC* Postoperative Pulmonary complication, *ITU* Intensive therapy unit, *LOS* length of stay, *VATS* video-assisted thoracosopic surgery

## Discussion

The objective of this Fit 4 Surgery app study was to develop, refine and examine the feasibility of an app that could deliver a pulmonary rehabilitation/exercise programme for operated lung cancer patients, in the comfort of their homes, safely and effectively. The fact that patients in the app group managed more sessions during the pre- and post-op period compared with the rehabilitation group, demonstrates that it is feasible to deliver the intervention and is acceptable and compliant to patients. Thirty one patients used the app and a separate 34 patients from the same surgical cohort attended exercise classes in the same period. No statistical comparisons have been made between groups but process measures data from both groups is presented to highlight issues in both types of rehabilitation.

The low number of rehabilitation classes attended prior to surgery highlights the difficulties of getting patients to classes. In the app group the system is efficient and effective at delivering exercise sessions despite a shorter time period to surgery. Furthermore, if we exclude the 3 poorly compliant patients with an exercise time of less than 10 min, the median total exercise time before surgery rises from 158 to 194 min. The drop-out rate of patients in the app group from restarting exercise after surgery was significantly lower than compared to class rehabilitation group (32% vs 79%). From the patient interviews, reasons for not continuing app-exercises after surgery were: pain, lack of motivation and generally feeling unwell. Further work is required in adapting the app to deal with the changing needs and motivation to exercise after surgery. To promote optimal and sustained behaviour change in physical activity, it is essential that interventions target recognised determinants and are theoretically grounded [11]. Behavioural change techniques (e.g., goal setting) underpinned by a sound theoretical basis need to be embedded within the app to improve efficacy [11].

Previous studies have shown a plethora of exercise interventions ranging from 12 to 20 classes (over 4 to 6 weeks) of combination strength and endurance exercise training and other adjuncts. Patients using the app in our study received on average 9 sessions which is less than the above studies but ISWT distances post rehabilitation before surgery increased by 99 m. This is above the minimally important clinically difference and is on par with other studies [12–14].

Attributing increases in ISWT results purely to exercise time captured by the app may be misleading as patients may have made broader lifestyle changes as other have described in other physical activity apps [15]. Thus, in subsequent studies it will be important to measure overall physical activity changes.

Physical activity interventions incorporating technology are actively being used in patients, mostly commonly with COPD, asthma and cardiovascular diseases [16–18]. Apps can successfully deliver patient education, disease self-management, and assist in the remote monitoring of patients [19, 20]. Low cost ‘off the shelf’ wearable technology (sensors) can track biometric data such as heart rate or steps and can be used in conjunction with apps to enable users to monitor their physical activity levels and progress. However, there is a shortfall of validated medical apps that present such sensor-based information to the user.

In one study, pulmonary rehabilitation was delivered using the LungFIT app on a smartphone. Heart rate and oxygen saturations were also measured using an integrated pulse oximeter [21]. However, the accuracy of the LungFIT physiological measurements were unreliable as the finger probe kept falling off during exercise. For the Fit 4 surgery study, we used a sturdy finger probe with a wrist strap attachment to ensure there were minimal movements during exercise to avoid spurious results. The Lung FIT app was tested in a healthy population; COPD and cardiovascular disease were exclusion criteria. Fit 4 Surgery app was tested in patients with multiple co morbidities. Furthermore, LungFIT was tested in a younger population (average age 47 years), consisting of researchers, family and friends, thus potentially creating bias. The LungFIT app was used in a laboratory environment and measurements collated by the researchers, not automatically or remotely as we have done in our study. Fit 4 surgery app was successfully delivered, in the patient’s home environment. There were no adverse or serious events, relating to using the app at home. Until now it was unclear as to whether a rehabilitation app is feasible in an older patient population in a home setting and our study shows that this is possible.

A major limitation of this feasibility study was that it was not powered to look at differences in clinical outcomes as the rehabilitation class cohort outcome measures were not comparable to the app group. Should further studies demonstrate this app makes a difference in clinical outcomes, we plan to make it freely available. The cost of implementation when this intervention is at the stage of being locally tailored and scaled up relates mainly to the extra amount of time a lung cancer nurse or physiotherapist would have to spend with the patient. From experience with our pilot study, we estimate that a total of 60 min allied health care professional contact time is required during the whole programme per patient. In a UK based system that would equate to a cost of between £16 to £34 per patient.

As this is a feasibility study, it has been helpful to identify potential problems allowing us make plans to mitigate them in future studies [22].

## Conclusion

In conclusion, the app would benefit from further development in terms of a more targeted and systematic incorporation of behavioural theory and techniques (such as goal setting) and individualised feedback (e.g. personal exercise goals met) to improve compliance. A broader randomised clinical trial with comparable patients to confirm clinical and wider patient lifestyle benefits of the further refined Fit 4 Surgery app is warranted.

## Additional file

Additional file 1: Users guide to Fit 4 Surgery app booklet. (ZIP 1123 kb)

## Data Availability

The datasets used and/or analysed during the current study are available from the corresponding author on reasonable request.

## Abbreviations

APP: Software application
BMI: Body mass index
COPD: Chronic Obstructive Pulmonary disease
DVD: Digital versatile disc
ECOG: Eastern cooperative oncology Group performance status
ISWT: Incremental shuttle walk test
ITU: Intensive therapy unit
mMRC: Modified medical research council dyspnoea scale
NHS: National Health Service
PPC: Postoperative pulmonary complications
QOL: Quality of Life
RESTful: Representational State Transfer
ROC: Rehabilitation for operated cancer
