# Supplementary material for: Fit 4 surgery, a bespoke app with biofeedback delivers rehabilitation at home before and after elective lung resection
Source: J Cardiothorac Surg. 2019 Jul 5;14:132. doi: 10.1186/s13019-019-0951-6 (PMC6611050; doi:10.1186/s13019-019-0951-6)
Supplement: Supplementary file 1 — Users guide to Fit 4 Surgery app booklet. (ZIP 1123 kb) [file 13019_2019_951_MOESM1_ESM.zip › Supplementary material legend.docx]

**Supplementary material 1 legend**

**Users guide to Fit 4 Surgery app booklet :** A. Screenshot showing how to turn on the iPad**.** B.Selecting the Fit for Surgery icon. C. Screenshot showing how to select an exercise from the list. D.Example of an exercise. E.One of the exercise videos showing feedback (e.g. heart rate and O2 saturation) and the option to stop if needed**.** F. Questions about why the exercise was stopped.

G. Selecting the severity of breathlessness. H. Confirmation of the accuracy of the information entered. I. Exercise summary screen which shows if target heartrate was achieved, average O2 saturation and motivation feedback. J. Screenshot showing the list of exercises, with information on the number of attempts for each one. K. Screenshot showing the option to stop or carry on with the session . L. Session summary screen showing the duration of exercise, average and maximum heart rate and O2 saturations achieved and motivation feedback. M. Screenshots showing feedback on the completed session. N. Additional comments box screenshot. O. Screenshot showing comparison with the previous session in terms of duration of exercise and oxygen saturations.
