# Supplementary material for: Fit 4 surgery, a bespoke app with biofeedback delivers rehabilitation at home before and after elective lung resection
Source: J Cardiothorac Surg. 2019 Jul 5;14:132. doi: 10.1186/s13019-019-0951-6 (PMC6611050; doi:10.1186/s13019-019-0951-6)
Supplement: Supplementary file 1 — Users guide to Fit 4 Surgery app booklet. (ZIP 1123 kb) [file 13019_2019_951_MOESM1_ESM.zip › Supplementary material 1R1.doc]

Ipad Guide

Firstly press the button at the bottom of the ipad. When the screen turns on drag you finger to the right across the screen

A. Screenshot showing how to turn on the iPad

Once the icons appear at the top of the screen select the Fit for Surgery icon

B. Selecting the Fit for Surgery icon

Following pressing the icon for Fit for Surgery a list of exercises will appear. If you drag your finger from the bottom of the screen towards the top you will have a choice of more exercises. Press the name of the exercises to start.

C. Screenshot showing how to select an exercise from the list

Once you have pressed the name of the exercise you would like to complete attach the heart rate monitor to your wrist and attach the rubber probe onto the end of your finger. After you have done this press start and watch the video on how the exercise is completed and complete the exercise for as long as you are able. We advise you aim to complete the exercise for approximately 5 minutes.

D. Example of an exercise

After you have completed 5 minutes of exercise or if you are too breathless to carry on press the stop button on the screen.

E. One of the exercise videos showing feedback (e.g. heart rate and O2 saturation) and the option to stop if needed

You will then be asked a number of questions about why you stopped and how breathless you are. Press next and the ipad will return to the list of exercises.

F. Questions about why the exercise was stopped

G. Selecting the severity of breathlessness

You will then be asked whether the answers you have given about your breathlessness are correct

H. Confirmation of the accuracy of the information entered

You will then be given a workout summery and given the option of being show how to make the exercise easier or harder. It is important to keep progressing your exercises and challenging yourself. Please then press next.

I. Exercise summary screen which shows if target heartrate was achieved, average O2 saturation and motivation feedback

After pressing next you will be taken back to then screen with the list of exercises. Please complete as many exercises as you feel able. When you have finished your exercise session please make sure you are pressing **Finish Session in the bottom right hand corner.**

J. Screenshot showing the list of exercises, with information on the number of attempts for each one

After you have pressed and session you will be asked whether or not you would like to stop this session or carry on. Perhaps have a go at completing one or two more exercises.

K. Screenshot showing the option to stop or carry on with the session

After completing your session you will be given a summery

L. Session summary screen showing the duration of exercise, average and maximum heart rate and O2 saturations achieved and motivation feedback

You will then be asked questions about your exercise session

M. Screenshots showing feedback on the completed session

You will be asked to rate you level of breathlessness and how difficult you found the exercises following the session

 M.Screenshots showing feedback on the completed session

You will then be given the opportunity to leave feedback either about the app or ways you think we may be able to improve it. You may wish to tell us if you did any other activities such as walking or cycling.

N. Additional comments box screenshot.

**The session you just completed will be compared to your last session completed. Then press next and you will return to the list of exercises.**

**O. Screenshot showing comparison with the previous session in terms of duration of exercise and oxygen saturations**
